# An Unusual Diagnostic Journey of a Spontaneously Resolving Breast Mass: When Rare Entities Cause Concerning Presentations

**DOI:** 10.7759/cureus.84924

**Published:** 2025-05-27

**Authors:** Junisha Martin, Sabrina Carpintieri, Jideka Nwosu, Elizabeth Kaur, Romane Joseph

**Affiliations:** 1 General Surgery, Ross University School of Medicine, Bridgetown, BRB; 2 General Surgery, American University of the Caribbean, Cupecoy, MAF; 3 General Surgery, Jackson North Medical Center, Miami, USA

**Keywords:** breast mass, cat scratch disease, diagnostic challenges, granulomatous inflammation, idiopathic granulomatous mastitis, inflammatory breast carcinoma, inflammatory breast lesion, multidisciplinary management, spontaneous resolution of lesions

## Abstract

This case report describes an atypical presentation of an enlarging breast mass over the course of 2.5 months in a 42-year-old African American woman, which initially raised suspicion for a serious condition but ultimately resolved spontaneously, emphasizing the importance of considering a broad differential diagnosis in complex breast pathologies. Her past medical history was significant for a similar mass in the same breast two years ago, which resolved spontaneously without medical intervention. Initial physical exam findings, including skin ulcerations and nipple retraction, raised suspicion for inflammatory breast cancer. The patient underwent a biopsy of the right breast and positive axillary lymph node. The biopsy revealed acute and chronic inflammation, granulation-type tissue, and focal granuloma with suppuration, but no evidence of malignancy. The discordance between the patient's physical exam findings and the biopsy results prompted the medical team to consider alternative diagnoses, such as idiopathic granulomatous mastitis and cat scratch disease. Due to the discrepancy, the patient was asked to return in one month for a repeat biopsy. However, upon her return, the breast mass and positive axillary lymph node had decreased in size significantly, with the ulcerated areas showing signs of healing. The medical team decided to forego the repeat biopsy and instead closely monitor the patient's condition. This case underscores the importance of considering a wide range of potential diagnoses, integrating clinical, radiological, and pathological findings, and adopting a multidisciplinary approach to ensure accurate diagnosis and appropriate management in complex clinical cases.

## Introduction

Inflammatory breast lesions present a diagnostic challenge due to their variable clinical presentations, which can range from benign conditions to malignant processes [1]. Idiopathic granulomatous mastitis (IGM) is a rare, benign chronic inflammatory breast lesion characterized by non-caseating granulomas and microabscesses in the mammary gland lobules [2]. Its clinical and imaging features often mimic breast cancer, particularly inflammatory breast cancer (IBC), making accurate diagnosis difficult [3].

IBC is an aggressive form of breast carcinoma that invades dermal lymphatics, causing inflammatory-like features such as warm, erythematous, and edematous skin, often with nipple inversion and dimpled or pitted skin, notably known as "peau d'orange" [4]. It accounts for 1-5% of all breast cancers globally and disproportionately affects younger women and certain racial groups [5,6]. Distinguishing features of IBC from other differential diagnoses, such as mastitis, include its rapid onset, skin changes such as dimpling or peau d'orange, lymphatic invasion, and lack of systemic signs like fever and chills. Early diagnosis is critical for prognosis, but its variable presentation can lead to underdiagnosis or misdiagnosis as other conditions like mastitis such as IGM or cat scratch disease (CSD) [7-9].

This case report highlights the importance of considering a broad differential diagnosis, including rare entities like IGM and CSD, when evaluating inflammatory breast lesions. It emphasizes the need for integrating clinical, radiological, and pathological findings to ensure accurate diagnosis and appropriate management in complex clinical presentations.

## Case presentation

We present the case of a 42-year-old African American woman with no significant past medical history who arrived at the surgical suite with an enlarging 4.5cm mass over 2.5 months in the right breast. The patient disclosed that two years ago, she experienced a lemon-sized lump in her right breast that persisted for approximately two months. The mass was accompanied by small, superficial ulcerations on the overlying skin and occasional discomfort. The mass was completely resolved during that time, and no medical treatment was sought. Six months later, the mass returned to the same location and has been increasing in size with ulcerations in the areolar region. The patient declined to obtain a mammography during that time due to the discomfort areolar lesion. Past surgical history consists of three prior cesarean sections (2011, 2015, 2021). This patient's prior surgical history and parity were relevant from a hormonal standpoint when evaluating her breast mass. It has been three years since her last pregnancy, during which she endorsed weaning in 2021 and denied any history of lactational mastitis. The patient denied any allergies, smoking, recent travel, pets at home, or any use of topical creams and ointments to the affected area. The patient denied any significant family or social history. 

The patient was fully alert and in no acute distress on physical examination. Patient vitals were obtained and reported normal (Table 1). Examination of the right breast revealed a mass in the areola measuring about 4.5cm with nipple retraction and a positive axillary lymph node. The right breast also showed cutaneous discoloration. The left breast appeared with no abnormalities.

**Table 1:** Patient vitals upon admission BPM, beats per minute; BRPM, breaths per minute.

Vital Signs Recorded	Recorded Values on Admission
Temperature	98.2 F
Blood Pressure	132/86 mmHg
Heart Rate	84 BPM
Respiratory Rate	19 BRPM
SpO2	99% on room air

The patient confirmed that the mass has increased over the last few months. Due to the findings, the patient was scheduled for an ultrasound-guided core biopsy of the right breast and right axillary lymph node due to the clinical scenario and high suspicion of malignancy versus fine-needle aspiration cytology. The core biopsy of the right breast at 12:00 revealed acute and chronic inflammation, granulation-type tissue, and focal granuloma with suppuration. It is important to note that the patient denied any exposure to pets, including cats. Immunohistochemical stains for pancytokeratin were negative for carcinoma. A Fite and Ziehl-Neelsen and GMS special stain was completed and remained negative for acid-fast and fungal organisms or bacterial rods. While we recognize the low sensitivity and limitations of Ziehl-Neelsen staining for Mycobacterium species, the staining approach was determined by the laboratory protocol. However, higher sensitivity, molecular testing was not utilized in this case. No evidence of malignancy was found in the biopsy of the right breast. A right axillary lymph node biopsy revealed sinus histiocytosis and acute nonspecific lymphadenitis but no evidence of malignancy. The patient returned to the office one week post-biopsy to discuss the results. During this visit, the patient reported feeling well with mild soreness at the biopsy site. Repeat physical examinations were still consistent with an enlarging areolar breast mass with skin ulcerations and nipple retraction. The right breast was moderately tender on palpation. Due to the discordance between the pathology report and the physical examination at this visit, a repeat ultrasound-guided biopsy with a clip of the right breast was highly recommended for further management. 

**Figure 1:**
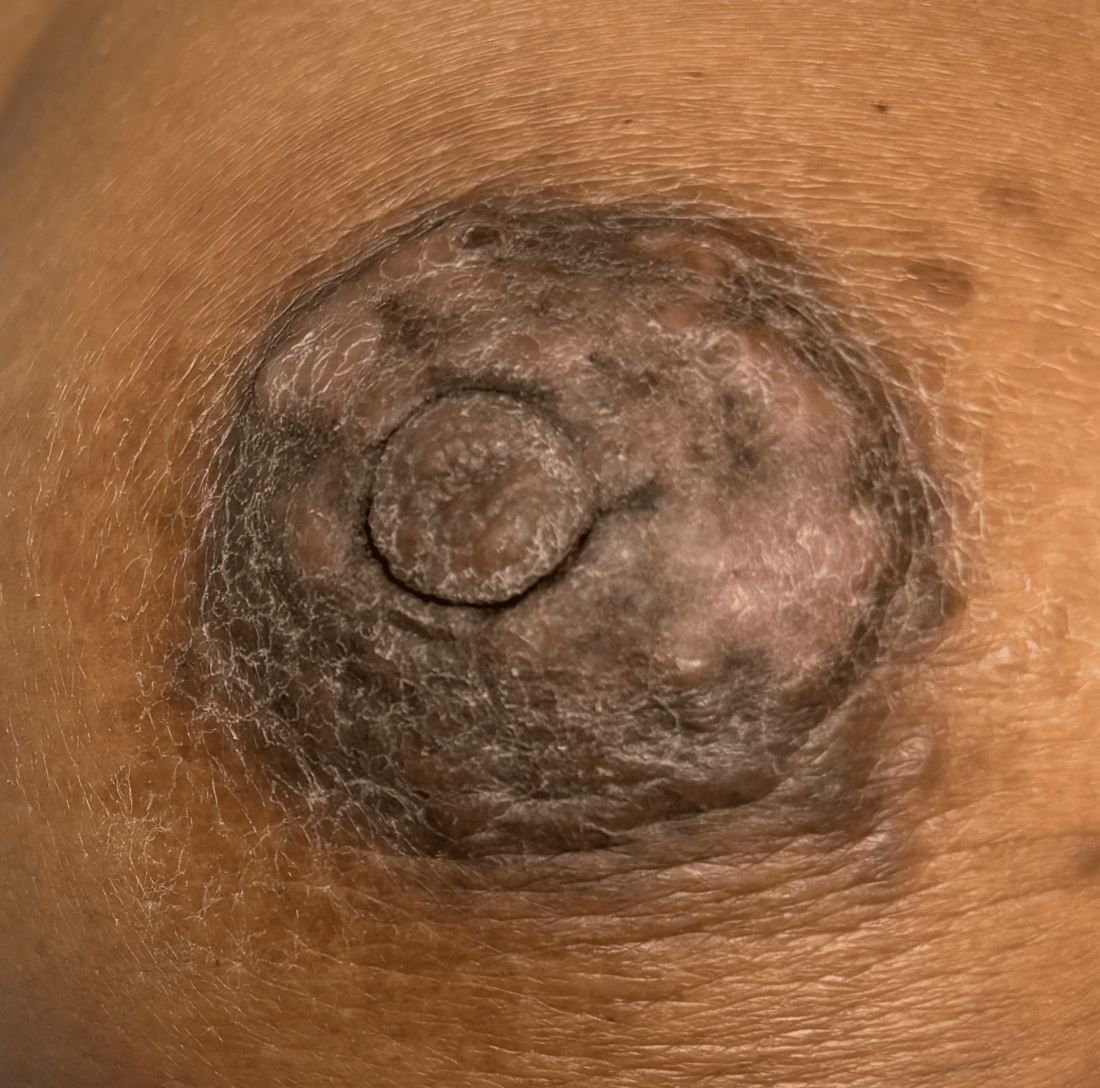
Right breast mass Recurring resolving right breast mass with discoloration.

The patient was scheduled for a repeat biopsy in one month. No medications were prescribed to the patient, and conservative measures were taken as we did not reach a final diagnosis. Upon return to the office to repeat the biopsy, the biopsy site was exposed, and physical examination revealed that the large right breast mass had decreased in size significantly. The mass was now measuring 3cm from 4.5cm and is non-tender, with minimal nipple retraction and healing ulcerations and scars with improving skin discoloration (Figure 1). The positive axillary lymph nodes have decreased in size significantly as well. The patient reported feeling well with no pain, soreness, or tenderness of the right breast. The left breast continued to appear with no abnormalities. The decreasing size of the lesion (mass effect), decreasing size of the lymph node, and the nature of the ulcer now healing ruled out IBC and made us lean toward an idiopathic vs inflammatory or infectious cause of this patient's presentation. The patient will follow up within one month or promptly if the lesion gets worse. We did not repeat the biopsy during that time. The above results and clinical progress were explained to the patient in great detail, and she verbalized understanding. We established a multidisciplinary plan of care for the patient in which she is to follow up with her primary care physician and surgeon for further management if there are signs of disease progression.

## Discussion

The presentation of this 42-year-old African American woman with a rapidly progressive breast mass, ulceration, nipple retraction, and axillary lymphadenopathy raised initial suspicion for IBC, a poorly characterized but aggressive type of breast cancer with an undefined mechanism driving its clinical phenotype, partially due to a lack of comprehensive large-scale genomic studies [10]. Despite its low incidence, IBC contributes to 7% of breast cancer-caused mortality [11,12]. It can sometimes mimic mastitis, and its aggressive nature means it can yield negative initial biopsies [13]. 

The core biopsy revealed acute and chronic inflammation, granulation-type tissue, focal granuloma with suppuration, and negative immunohistochemical stains for carcinoma, along with negative special stains for acid-fast organisms, fungal elements, and bacterial rods, which argued against a malignant or infectious process. Molecular staining and culture were not performed due to the standard laboratory protocol. We did not have influence on the selection or availability of advanced diagnostic modalities. However, these findings instead raised the possibility of alternative diagnoses such as IGM, a rare and poorly understood condition usually diagnosed by exclusion, typically seen in women of childbearing age.

IGM presents as a painful, scarring condition with inflammatory nodules of the breast. Its clinical appearance can easily be misdiagnosed as breast abscess, furuncles, or cellulitis. However, furuncles and cellulitis tend to have a diffuse distribution along the breast skin and subcutaneous tissue rather than a distinct mass-like lesion as seen in this patient. Some cases resolve spontaneously without treatment or with the use of antibiotics if an infection is suspected, while others can persist for long time periods [14]. The potential for this rare condition to clinically resemble an infectious process likely contributed to the initial diagnostic complexity in this particular case, despite the patient ultimately not receiving a definitive diagnosis of IGM.

Furthermore, although the patient denied cat exposure, CSD caused by Bartonella henselae, known to manifest with skin lesions and regional lymphadenopathy mimicking other breast pathologies, could not be definitively excluded based on the initial biopsy report. CSD is a zoonotic infection typically transmitted to humans when an infected cat's saliva comes into contact with an open wound via a cat scratch, bite, or the cat licking an open wound [14]. While lymphadenopathy is the most common presentation, CSD can also involve various organs and tissues, including the breast. CSD can present as mastitis or disguise itself as a solitary breast mass. Diagnosing CSD typically involves serological testing for Bartonella antibodies, along with characteristic histopathological findings in the lymph nodes, a history of animal exposure, and ruling out other potential causes of lymphadenopathy through additional laboratory investigations [15]. The initial biopsy findings of granulomatous inflammation raised suspicion for CSD in this patient, even though special stains were negative for identifiable infectious organisms or patient exposure. Due to negative exposure, negative stains, and no clinical signs of infection in this patient, no oral or intravenous antibiotics were administered.

There is no gold standard to diagnose CSD, and it is difficult to culture. It can be detected via serologic or polymerase chain reaction (PCR) methods [16]. Similar to IGM, false-negative results on tissue biopsies are common with CSD due to the fastidious nature of Bartonella, which requires specialized culture methods or molecular testing. Despite the patient's denial of cat exposure, CSD remained a plausible consideration due to the clinical and pathological findings in this case and a possible false-negative on the obtained biopsy. A definitive diagnosis may require advanced testing such as Bartonella-specific serologies, PCR assays, or immunohistochemical stains [16], which were not completed in this study. The potential for CSD to mimic various breast pathologies and malignancies further highlights the importance of considering a wide range of potential diagnoses and pursuing appropriate diagnostic evaluation when clinical and pathological findings conflict.

The subsequent clinical improvement, with spontaneous drainage, resolving ulcer, and decreasing size of the mass and lymph nodes after conservative management, supported an idiopathic vs inflammatory or infectious process rather than a malignancy. While a repeat biopsy was considered, the resolving nature of the lesion prompted close clinical monitoring instead. The false negative rate for the first core needle biopsy in IBC is 2-9% [13], which creates a low threshold for repeat biopsy. We did not deem it necessary to repeat the biopsy with this patient's clinical presentation. 

Coordinating input from various medical disciplines, including primary care physicians, surgeons, infectious disease or oncology experts, is vital for reaching an accurate diagnosis and providing comprehensive treatment. This multidisciplinary approach becomes especially important if the breast lesion persists or recurs, as additional diagnostic tests or therapeutic procedures may be warranted. This case highlights the importance of considering a broad differential diagnosis and integrating clinical, radiological, and pathological findings in the evaluation of breast masses to avoid unnecessary aggressive treatments and ensure appropriate management.

## Conclusions

This case illustrates the diagnostic challenges of inflammatory breast lesions with overlapping clinical presentations. While initial findings suggested IBC, biopsy results were negative for malignancy, pointing toward a benign inflammatory or infectious etiology, such as IGM or CSD. The conflicting examination findings (decreasing mass size, resolving ulceration, and nipple retraction) and negative biopsy emphasize the importance of maintaining a broad differential diagnosis on initial presentation. Rare entities like these can mimic malignancies or mastitis. The spontaneous improvement after conservative management provided further evidence against a malignant process. No therapeutic interventions for CSD were taken due to low risk factors and susceptibility, and no additional positive lymph nodes were found. A repeat biopsy was not warranted with the low false-negative rate for core-needle biopsy. This case reinforces the need for interdisciplinary collaboration, and further research into inflammatory breast pathologies is vital. Maintaining an open perspective and integrating all available data are essential when evaluating breast masses, facilitating timely diagnosis, and preventing unnecessary aggressive treatments. This case concluded without a definitive diagnosis, highlighting the importance of flexible management guided by clinical improvement in complex presentations.
